# Poor Dental Status and Oral Hygiene Practices in Institutionalized Older People in Northeast Brazil

**DOI:** 10.1155/2009/846081

**Published:** 2009-05-26

**Authors:** Luciene Ribeiro Gaião, Maria Eneide Leitão de Almeida, José Gomes Bezerra Filho, Peter Leggat, Jorg Heukelbach

**Affiliations:** ^1^Department of Community Health, School of Medicine, Federal University of Ceará (UFC), Fortaleza, Ceará 60430-140, Brazil; ^2^Department of Clinical Dentistry, School of Pharmacy, Dentistry and Nursing, Federal University of Ceará (UFC), Fortaleza, Ceará 60430-140, Brazil; ^3^School of Public Health, Tropical Medicine and Rehabilitation Sciences, James Cook University, Townsville 4811, Australia; ^4^School of Public Health, University of the Witwatersrand, Johannesburg 2050, South Africa

## Abstract

In this study we describe the dental status and oral hygiene practices in institutionalized older people and identify factors associated with poor dental status. A cross-sectional study was performed in a nursing home in Fortaleza, the capital of Ceará State (northeast Brazil). The number of decayed, missing, and filled teeth (DMFT) was assessed in the residents of the nursing home (*n* = 167; mean age = 76.6 years). The mean DMFT value was 29.7; the mean number of missing teeth was 28.4. Ninety-three (58.1%) were edentulous. Almost 90% practiced oral hygiene, but only about half used a toothbrush. Only 8% had visited a dentist in the preceding three months. Most of the variables regarding oral hygiene habits (such as the use of toothbrush, frequency of oral hygiene per day, regular tooth brushing after meals) did not show any significant association with the DMFT. In multivariate regression analysis, age, general literacy level, and practice of oral hygiene were independently associated with the DMFT (R^2^ = 0.13). Institutionalized older people in northeast Brazil have poor dental status, and oral hygiene practices are insufficient. Dental health education is needed focusing on the special needs of this neglected and socioeconomically deprived population to improve their quality of life.

## 1. Introduction

Throughout the world, the proportion of older people in the general population has increased in recent years. In addition to other general health issues there are tremendous implications for oral health. In older people there is a high level of tooth loss, dental caries, periodontal disease, xerostomia and cancer [1, 2]. In addition, poor oral health and poor general health are often interrelated. 

There are several studies on the dental status of institutionalized older people throughout the world. For example, studies have been reported from the United States [3], Australia [4, 5], Canada [6], India [7], Italy [8, 9], Greece [10], Croatia [11], the Fiji Islands [12], Hong Kong [13] and Singapore [14], indicating that the dental status of institutionalized older people is generally poor, with a high decayed, missing, or filled teeth (DMFT) index. 

In Brazil, the demographic transition has been particularly accelerated, which has been associated with a higher demand for long care facilities for older people. Despite this, data on institutionalized older people from Brazil are scarce. Some reports from south Brazil suggest that the oral health of institutionalized older people is poor [15, 17]. Data on the dental status of institutionalized older people from the northeast of the country, which has different socioeconomic and cultural characteristics from the south and is considered the poorest region of the country, are virtually nonexistent. In particular, there is no data on the factors that contribute to poor oral health status in these special populations and the state of oral hygiene practices. To assist in filling this gap, a cross-sectional study was performed assessing oral status and hygiene practices of residents in a nursing home in Fortaleza, a capital city in northeast Brazil.

## 2. Methods

This cross-sectional study was performed in the institution “Lar Torres de Melo”, a nursing home in Fortaleza, capital of Ceará State in northeast Brazil. Fortaleza has a population of approximately 2,4 million people. “Lar Torres de Melo” is the largest institution for older people in the State and typically would have about 220 residents. All individuals residing in the institution and who matched the WHO definition for older people were eligible for the study. Of the 220 residents, 167 (75.9%) met this inclusion criterion of ≥65 years of age. 

The study consisted of a clinical oral examination; a structured interview using a pretested questionnaire to assess oral hygiene practices, such as frequency of oral hygiene, type of hygiene, and visits to dentists; the analysis of the patients' records to obtain data on age, marital status, time in the institution, general literacy level (years of schooling), and retirement status. Professional carers were interviewed in some cases, particularly when the older people were not in the physical or mental condition to answer questions. The clinical oral examination consisted of assessment of the DMFT using Brazilian standard procedures, which were similar to WHO standards [18]. 

To reduce interobserver bias, all clinical examinations were done by a single investigator, an experienced dentist. Detailed clinical data, including periodontal diseases and the use and need of prosthesis of the study population, are published elsewhere [19].

Data were double entered into a database using the Epi Info software package (Version 6.04d, Centers for Disease Control and Prevention, Atlanta, USA) and checked for entry errors. After bivariate analysis using t test and Kruksal Wallis test, appropriate multiple linear regression, using the DMFT as the dependent variable, was performed. All variables with a significance level of *P* < .25 were included in the regression analysis. Backward elimination was used. Regression analysis was done with STATA^TM^ software (Version 8; Stata Corporation, College Station, Tex, USA). The study was approved by the Ethical Review Board of the Federal University of Ceará, Brazil. Informed written consent was obtained from study participants or the appropriate legal guardians.

## 3. Results

One hundred and sixty individuals were included in the study (95.8% of the target population). The mean age was 76.6 years (standard deviation = 8.6 years), with a maximum age of 100 years. The mean time of living in the institution was 8.2 years (range: 1 month–50 years). Many were illiterate. Demographic and socioeconomic variables are detailed in Table 1.

Ninety-three (58.1%) were edentulous, and the mean number of teeth missing was 28.4. The mean DMFT of all study participants was 29.7 (SD = 4.4; edentulous participants were allocated a DMFT of 32). The mean DMFT for the 67 dentate individuals was 26.6 (SD = 5.3); 16 (23.9%) of these presented a DMFT of 32. Of all participants, 109 (68.1%) had a DMFT of 32, which meant that they did not have a single healthy tooth. 

Oral hygiene practices are detailed in Table 2. Almost 90% practiced oral hygiene, and more than three-quarters of these did not need the help of a professional carer. Only about half of the study participants used a toothbrush. A minority had visited a dentist in the preceding three months, most commonly for dental extraction. Only 58 participants perceived a need for treatment; however, clinical examination revealed the need for at least some dental treatment in all 160 cases.

In the bivariate analysis, only sex, age, and general literacy level were significantly associated with the DMFT (Table 3). Females presented with a higher DMFT. However, this variable was confounded by age (i.e., the mean age of females was higher than that of males). In the multiple regression analysis, age (*β* = 0.11; *p* = .005), general literacy level (*β* = −0.79 for each additional year of schooling; *P* = .003), and practice of any type of oral hygiene (*β* = 2.5; *P* = .02) were independently significantly associated with the DMFT. However, only 13% of the variance of the DMFT could be accounted for by these three independent variables (*R* ^2^ = 0.13).

A lower DMFT was observed among individuals who performed their own oral hygiene and used a toothbrush. However, these and other variables regarding oral hygiene habits (such as practicing any type of oral hygiene, frequency of oral hygiene, cleaning the tongue, and regular tooth brushing after meals) did not show any significant association with the DMFT.

## 4. Discussion

Our study shows that the experience of dental caries (as represented by the DMFT index) in institutionalized older people in northeast Brazil is extremely high. The data also found that there were insufficient oral hygiene practices in this special population. Similar to most studies on institutionalized older people throughout the world [3–14], a high proportion of the population was edentulous.

The oral health status in the general older people population has been addressed increasingly in the past years, but the oral health of institutionalized older people and underprivileged populations continues to be a neglected issue [1, 20–23]. The DMFT varies considerably not only between different socioeconomic groups, but also between different countries and cultures, and it is consistently higher in institutionalized people than among community-dwelling populations [4–15, 17, 24]. For example, in Hong Kong, the mean DMFT index was 21.4 in institutionalized as compared to 17.7 in noninstitutionalized older people [24]. A small number of other studies from southern Brazil indicate that the oral health status of institutionalized as well as noninstitutionalized older people was also very poor [15, 17, 25]. In southern Brazil, the DMFT in institutionalized was as high as 30.8 [15], which was similar to our study population from the northeast (DMFT = 29.7). A representative national survey made in 2002/2003 on oral health conditions in the Brazilian population revealed a mean DMFT of 27.8 in community-dwelling older people [18].

Oral hygiene practices in the study population were inadequate, and this group of older people had poor access to dental health care. In all residents, a need for treatment by a dentist was detected by the clinical assessment, but only 44% had perceived that need. In Brazil, residents in these institutions tended to have very poor socioeconomic conditions, and the findings of the present study indicate that dental health care for the poorest and oldest in society needs more attention. Initially, these issues were discussed with the director of the institution, and a dentist now regularly performs preventive and curative oral health care activities. Residents also received free treatment after the study.

On the public health level, health education programs focusing on the special needs of these populations are mandatory. An integrated approach is needed, and oral health education should include all stakeholders [26]. Additionally, it is necessary to implement curative and rehabilitation measures in these populations to reduce the need for future dental treatment [27]. 

Due to the cross-sectional design, causal and temporal relationships between independent variables and DMFT could not be clearly established. In addition, a possible limitation of the study was that it was conducted in a single institution, although this was the largest nursing home in the state of Ceará. 

In conclusion, this study shows that the dental status of institutionalized older people in the state of Ceará is very poor. There is a lack of perceived need for dental services and of adequate oral self-care. Dental health education is also needed focusing on the special needs of this neglected and socioeconomically deprived population to improve their quality of life.

## Figures and Tables

**Table 1:** Demographic and socioeconomic characteristics of institutionalized older people in northeast Brazil.

	Total N (%)*	Dentate N (%)*	Edentulous N (%)*	*P* value (dentates versus edentulous)
Sex				
Male	81 (50.6%)	43 (53.1%)	38 (46.9%)	*P* = .004
Female	79 (49.4%)	24 (30.4%)	55 (69.6%)	
Age				
65–74 years	75 (46.9%)	33 (44.0%)	42 (56.0%)	*P* = .7
75–84 years	56 (35.0%)	21 (37.5%)	35 (62.5%)	
≥85 years	29 (18.1%)	13 (44.8%)	16 (55.2%)	
Marital status				
Single/widowed/divorced	141 (88.1%)	54 (38.3%)	87 (61.2%)	*P* = .01
Married	19 (11.9%)	13 (68.4%)	6 (31.6%)	
Time in institution				
0–5 years	85 (53.1%)	42 (49.4%)	43 (50.6%)	*P* = .1
6–10 years	32 (20.0%)	12 (37.5%)	20 (62.5%)	
>10 years	43 (26.9%)	13 (30.2%)	30 (69.8%)	
General literacy level				
Illiterate	65 (40.6%)	26 (40.0%)	39 (60.0%)	*P* = .7
Literate	95 (59.4%)	41 (43.2%)	54 (56.8%)	

*% in “total” column are column percentages; % in “dentate” and “edentulous” columns are row percentages.

**Table 2:** Oral hygiene practices of institutionalized older people in northeast Brazil (**n** = 160).

	Total N (%)	Dentates N (%)	Edentulous N (%)	*P* value (dentates versus edentulous)
Practices any type of oral hygiene				
No	17 (10.6%)	5 (7.5%)	12 (12.9%)	*P* = .3
Yes	143 (89.4%)	62 (92.5%)	81 (87.1%)	
Who performs the oral hygiene?^†^				
Patient	111 (77.6%)	49 (79.0%)	62 (76.5%)	*P* = .7
Carer	32 (22.4%)	13 (21.0%)	19 (23.5%)	
Use of a toothbrush				
No	78 (48.7%)	33 (49.3%)	45 (48.4%)	*P* = .9
Yes	82 (51.3%)	34 (50.7%)	48 (51.6%)	
Received health education regarding oral hygiene*				
No	54 (34.2%)	27 (40.3%)	27 (29.0%)	*P* = .16
Yes	104 (65.8%)	40 (59.7%)	64 (68.8%)	
Regular toothbrushing after meals				
No	70 (43.8%)	37 (55.2%)	33 (35.5%)	*P* = .01
Yes	90 (56.2%)	30 (44.8%)	60 (64.5%)	
Frequency of oral hygiene per day				
Never	17 (10.6%)	10 (14.9%)	7 (7.5%)	*P* = .4
Once	41 (25.6%)	15 (22.4%)	26 (28.0%)	
Twice	66 (41.3%)	26 (38.8%)	40 (43.0%)	
Three times or more	36 (22.5%)	16 (23.9%)	20 (21.5%)	
Cleaning the tongue				
No	85 (53.1%)	39 (58.2%)	46 (49.5%)	*P* = .3
Yes	75 (46.9%)	28 (41.8%)	47 (50.5%)	
Visit to a dentist in the last 3 months*				
No	144 (91.7%)	56 (83.6%)	88 (94.6%)	*P* = .1
Yes	13 (8.3%)	8 (11.9%)	5 (5.4%)	
Reason for the last visit to dentist*				
Toothache	8 (7.1%)	5 (7.5%)	3 (3.2%)	—
Routine exam	5 (4.5%)	1 (1.5%)	4 (4.3%)	
Dental extraction	43 (38.4%)	23 (34.3%)	20 (21.5%)	
Need of prosthesis	33 (29.5%)	6 (8.9%)	27 (29.0%)	
Perceived need for treatment*				
No	73 (55.7%)	23 (34.3%)	50 (53.8%)	*P* = .006
Yes	58 (44.3%)	32 (47.7%)	26 (27.9%)	

*data were not available in all cases; ^†^of those practicing oral hygiene.

**Table 3:** Bivariate analysis of variables associated with the number of decayed, missing, and filled teeth (DMFT) in institutionalized older people in northeast Brazil.

Variable	Mean DMFT	Standard deviation	*P*-value
Sex			
Male	29.0	5.10	.03
Female	30.5	3.31	
Age (years)			
65–74	28.7	5.27	.05
75–84	30.3	3.64	
≥85	31.5	1.12	
Marital status			
Single/widower/divorced	29.7	4.36	.91
Married	29.6	4.45	
Time in institution			
0–5 years	29.2	4.88	.17
6–10 years	30.0	3.97	
>10 years	30.5	3.39	
General literacy level			
Illiterate	30.8	3.14	.03
Literate	29.6	4.14	
Practices any type of oral hygiene			
No	28.4	5.07	.24
Yes	29.9	4.25	
Who does the oral hygiene			
Older people	29.6	4.48	.08
Carer	30.9	3.24	
Use of tooth brush			
No	30.4	3.69	.06
Yes	29.1	4.84	
Received health education on oral hygiene			
No	29.0	5.35	.18
Yes	30.0	3.75	
Regular tooth brushing after meals			
No	29.1	4.59	.12
Yes	30.2	4.13	
Frequency of oral hygiene per day			
None	28.4	5.07	.41
Once	29.8	4.05	
Twice	30.5	3.25	
Three times or more	28.8	5.76	
